# Artificial Intelligence in Point-of-Care Ultrasound: Domains, Barriers and a Framework for Future Development

**DOI:** 10.1016/j.acepjo.2026.100491

**Published:** 2026-08-19

**Authors:** Robinson M. Ferre, Rachel B. Liu, HF Samuel Lam, Michael Blaivas, Laura Oh, Rohit B. Sangal, Cristiana Baloescu, Srikar Adhikari

**Affiliations:** 1 Department of Emergency Medicine, Indiana University School of Medicine, Indianapolis, Indiana, USA; 2 Department of Emergency Medicine, Yale School of Medicine, New Haven, Connecticut, USA; 3 Department of Pediatrics, University of Colorado School of Medicine, Aurora, Colorado, USA; 4 University of South Carolina School of Medicine, Columbia, South Carolina, USA; 5 Department of Emergency Medicine, Emory University School of Medicine, Atlanta, Georgia, USA; 6 Department of Emergency Medicine, University of Arizona College of Medicine, Tucson, Arizona, USA

**Keywords:** ultrasonography, artificial intelligence, machine learning, workflow integration, diagnostic imaging

## Abstract

The use of artificial intelligence (AI) within medicine has increased dramatically in the past few years, and applications for point-of-care ultrasound (POCUS) have followed a similar trend. Although physicians believe that POCUS AI applications have the potential to improve clinical practice, adoption of current applications remains limited. A lack of outcomes-based evidence, bias within datasets, poor assimilation within current workflows, and regulatory uncertainty are some of the major barriers that lead to poor adoption rates. To improve integration, stakeholders (physician leaders, researchers, health care executives, and industry partners) should begin any new POCUS AI development project by first examining the different domains where POCUS AI applications are most needed, including education, clinical practice, workflow, research, and administration. A gap analysis with clearly defined outcomes should come next, followed by an examination of how the new POCUS AI application will integrate into existing workflows. Training data sets that are reflective of real-world scenarios, including limitations encountered by end users, are essential. POCUS AI applications that integrate with existing workflows, have explainable outputs, and have been codeveloped with end users will improve adoption. Although regulatory pathways are evolving, engaging regulators early in the process and identifying viable reimbursement pathways are key strategies that will improve both the development and adoption of POCUS AI applications in the future.

## 1 Introduction

The use of artificial intelligence (AI) has become increasingly integrated into the clinical practice of emergency medicine (EM), with notable expansion in the area of point-of-care ultrasound (POCUS).^1^, ^2^, ^3^ Ultrasound equipment marketed and sold for POCUS users has progressively incorporated AI-enabled tools that guide image acquisition,^4^ perform automated measurements, such as left ventricular ejection fraction or bladder volume,^5^^,^^6^ and identify key sonographic findings, such as B lines or free fluid.^7^, ^8^, ^9^

Despite the proliferation of AI technology in POCUS, current literature has not clearly demonstrated a positive impact of POCUS AI in clinical care outcomes.^10^ It remains unclear if AI could improve diagnostic accuracy, provide useful additional information to guide patient management, or contribute to more efficient utilization of resources in real-world settings. Likewise, the impact of AI on POCUS educators, learners, and administrators has not been adequately explored.

In this manuscript, we describe 5 chief domains where AI can be integrated into POCUS training and clinical care. We propose a framework for stakeholders involved in future POCUS AI development, including POCUS physician leaders, researchers, industry representatives and health care executives. We link the 5 unique POCUS AI development domains with the major barriers to adoption. We propose a practical set of development recommendations and guardrails for future POCUS AI tools. Although this article is written using an EM lens, we believe the concepts discussed are relevant to POCUS in other medical specialties.

## 2 POCUS AI Domains

Current practice of POCUS encompasses image acquisition, documentation of image interpretation, training users to perform and incorporate POCUS into medical decision-making, program administration, and clinical research.^11^ To better characterize how AI products can enhance POCUS, leaders from the American College of Emergency Physicians (ACEP) Emergency Ultrasound Section’s Industry Round Table developed a framework to describe a set of domains where AI could be used within the field of POCUS. Subsequently, members of the ACEP Emergency Ultrasound Section were surveyed about this framework in 2022. The results from the survey were used to refine the key domains in which AI could enhance POCUS for clinical users, educators, and administrators.^12^ The final 5 domains chosen were education, clinical practice, workflow, research, and administration (Table 1).

**Table 1:** Domains for development of artificial intelligence programs for point-of-care ultrasound.

POCUS development domain	Description	Targeted end-user	Examples of AI program utility
Education	Tools used by learners to facilitate and augment learning. This is particularly relevant when learning to obtain and optimize image acquisition.	Learners at beginning, intermediate and advanced stages. Educators developing POCUS curricula who may incorporate different AI tools throughout the learning process.	1. Relevant sonographic anatomy is displayed on the screen during image acquisition to assist and reinforce sonographic anatomy for new learners 2. Probe guidance may be suggested to optimize visualization of required structures
Clinical	Tools to assist the clinician to efficiently and effectively answer clinical questions and interpret the exam. This area may incorporate classification or prognostication of disease severity	Clinician performing POCUS for clinical care	1. Exam-specific measurements (e.g., bladder volume, left ventricular ejection fraction, etc.) 2. Detection of exam specific abnormalities (e.g., B-lines, free fluid, etc.)
Workflow	Tools to improve completion of all required elements of an exam (e.g., patient demographics, saved images with appropriate labels)	Clinician performing POCUS for clinical care	1. Reminders to add required patient demographics 2. Auto-labeling exam-specific views 3. Auto-saving images or cine clips
Research	Tools to assist with POCUS research. This may span several domains within the use of POCUS, such as image acquisition, interpretation and clinical integration	Researcher	1. Sort and analyze POCUS studies based on indication, user, finding, etc. 2. Compare POCUS findings based on other data sources (e.g., EHR, PACS, etc.)
Administrative	Tools to assist with running a POCUS program (e.g., quality assurance, reports and dashboards)	POCUS Director, Fellowship Director, QA Reviewer	1. Assist with the process of performing QA on POCUS exams 2. Assign studies for QA review based on pre-determined parameters 3. Create reports of users who are not compliant with POCUS documentation requirements

POCUS, point-of-care ultrasound; QA, quality assurance; EHR, electronic health record.

These 5 domains act as a framework to identify opportunities for POCUS AI development. Identifying gaps within each domain is a crucial first step in the development of effective and user-centered AI solutions. By examining each domain individually, POCUS AI developers are more likely to find cost-effective, impactful, and easily adoptable solutions that solve current problems encountered by POCUS users, educators, researchers, and administrators.

## 3 Current Pocus AI Program Challenges and Shortcomings

The adoption of POCUS AI tools remains limited, even though the majority of clinicians, when asked, believe that AI can improve POCUS practices.^13^ Clinicians prefer AI tools that are easy to use, offer real-time decision support, and reduce cognitive load, but few ultrasound vendors on the market offer this full-scale capability.^14^, ^15^, ^16^ Educators seek AI tools for competency assessment and adaptive learning, but few products provide validated educational feedback.^17^ Researchers need access to large, standardized datasets to compare AI algorithms and reduce bias, but proprietary data remains siloed.^16^ Administrators seek quality-assurance metrics, billing support, and workflow efficiency, but AI integration with hospital systems is lacking.^13^ Patients expect equitable care and improved outcomes, but few AI POCUS products have taken these factors into consideration in their development.^16^ Although the factors affecting adoption are complex, we posit that the following 5 factors are the main barriers to POCUS AI development and adoption.

### 3.1 Technology-driven solutions, instead of outcome-driven solutions

Many current POCUS AI tools are developed around technical capabilities like improved image quality or quicker measurements, rather than clearly defined clinical or operational problems.^16^ For instance, a commercial nerve-identification tool uses deep learning to segment brachial plexus structures, achieving recognition accuracy >88%, yet it is explicitly marketed only for visualization, not for guiding interventions.^18^^,^^19^ Most current POCUS AI tools are developed around technical capabilities.^1^ Although this leads to the development of enhanced features, they lack a more rigorous outcome-driven approach to solving real-world problems. A problem-driven approach that includes measurable reductions in diagnostic error, mitigation of workflow issues, or scalable training outcomes is more likely to produce POCUS AI products that are needed and more readily adopted by end users. Emerging AI-guided acquisition and education platforms demonstrate that training-focused solutions are feasible, but robust, outcomes-based, problem-driven designs remain the exception rather than the norm.^20^^,^^21^

### 3.2 Data limitations and bias

POCUS images are notoriously noisy and vary with patient habitus, transducer type, environment, and operator skill.^16^ In addition, ultrasound data have less defined edges and require task-specific models. Mathematical analysis of ultrasound imagery compared with other medical images (e.g., CT) has shown that ultrasound gray-scaled images have much less context variation when individual pixels are evaluated throughout an entire image, indicating a need for different AI algorithms than might be optimal for other image types as well as larger training datasets.^22^ Many AI models for POCUS are trained on small, homogeneous datasets that can introduce demographic bias and poor generalization.^16^^,^^23^ Bias may be introduced during data collection, annotation, or model optimization.^24^ Bias may also arise when models are trained solely on high-quality images. Evidence suggests that AI systems must learn from images acquired by users with different levels of expertise.^16^ Otherwise, they may fail due to poor technique or “domain shift.” In addition, performance degradation in unseen environments underscores the need for diverse, representative datasets and continual local fine-tuning.^25^

This domain shift limitation of AI imaging algorithms is increasingly recognized as problematic by regulatory bodies and must be addressed by developers.^26^ Furthermore, current products rarely disclose dataset composition or sources of bias, making it difficult for users to judge reliability. As indicated by the COMPASS-AI survey, an international survey of 1154 health care professionals that examined perceived barriers regarding POCUS AI, clinicians worry about the “black-box” nature of AI with a lack of explainability that undermines trust.^13^

### 3.3 Integration, workflow, and training barriers

Current POCUS AI tools are rarely integrated into existing clinical workflows. The COMPASS-AI survey found that this gap, along with limited electronic health record (EHR) compatibility, was a major barrier to adoption.^13^ The need for vendors to create a comprehensive ecosystem-like AI solution weighs heavily on business decisions regarding AI POCUS development. At present, clinicians must purchase stand-alone AI applications that may be disjointed and cumbersome to integrate. The need for multiple applications leads to rapid accumulation of costs, while insufficient technical support at both the vendor and local level limits scalability.^1^^,^^27^ As POCUS workflow differs from other imaging modalities, since acquisition and interpretation occur simultaneously, AI tools must operate seamlessly without adding steps to the workflow process.

Training and human-factor issues further impede adoption. Clinicians report that they lack adequate training for AI-assisted ultrasound tools and are concerned that AI errors may lead to incorrect diagnoses.^13^ Currently, almost half of clinicians report that they would verify every AI-generated finding before making a clinical decision, a higher rate than reported for other imaging modalities (e.g., chest x-ray).^13^ Cultural resistance and absence of institutional guidelines are also recognized barriers. Even when AI improves efficiency, the learning curve and the perception of AI as a threat to professional autonomy may also reduce adoption.^13^

### 3.4 Lack of prospective clinical validation and outcome evidence

Beyond the issue of technology-driven development described above, even AI tools with strong technical performance often lack prospective clinical evidence demonstrating meaningful improvements in diagnostic accuracy, clinical decision-making, or patient outcomes in real-world environments. For instance, small, single-site studies have demonstrated that deep learning models can detect free fluid in Morison’s pouch with high sensitivities ranging from 83% to 98% and excellent specificities ranging from 93% to 100%.^28^, ^29^, ^30^ In another instance, Narang et al. demonstrated that a small group of nurses with no ECHO training could use AI guidance to acquire diagnostic-quality cardiac views of left ventricle size and function with > 98% success.^19^ Despite the promise of these small studies, prospective multicenter trials evaluating how these tools work in a fast-paced clinical environment, affect clinical decision-making, treatment times, or patient morbidity and mortality are scarce.^1^^,^^31^ Clinicians report the absence of clinical validation as one of the top barriers to adoption. Without it, administrators and payers have little incentive to invest.^13^

### 3.5 Regulatory and ethical uncertainties

With the rapid emergence and evolution of AI in medical imaging, regulatory pathways are often behind and struggle to keep pace.^26^^,^^32^ Clinicians desire regulatory approval by government agencies and strong evidence before being willing to adopt AI for medical decision-making.^13^ In addition, AI tools also raise medicolegal issues around who is responsible for errors, how the standard of care evolves, and how transparently AI use is communicated to patients.^33^^,^^34^ At present, primary liability lies with the clinician; however, institutions that deploy AI tools and vendors whose designs or training data are shown to be defective may also share responsibility with clinicians and health care organizations.^33^^,^^34^ Proposed policy approaches such as “safe harbor” frameworks have been suggested to clarify liability when clinicians interact with AI tools but have been largely conceptual and not broadly implemented yet. These uncertainties create challenges to both health care organizations and clinicians who may otherwise be early adopters of these new technologies.

Each of these barriers affects the 5 POCUS AI development domains differently (Table 2). Although some barriers, such as workflow integration and regulatory requirements, are common across multiple domains, others are domain-specific. For example, data bias and outcome validation are crucial for clinical POCUS AI tools, whereas data standardization is more relevant to research applications. Identifying the most relevant barriers for each domain is essential for prioritizing development efforts and facilitating successful implementation.

**Table 2:** POCUS AI development barriers by domain.

POCUS development domain	Development barriers	Significance
Education	Outcome measures and integration	Limited evidence that AI-assisted training tools improve learning outcomes or enhance POCUS competency.
Clinical	Outcome-driven validation, imaging data limitations, workflow integration, regulatory and ethical challenges	Clinical POCUS AI tools face the greatest number of development barriers. A clear development roadmap is essential to systematically address these challenges and support successful clinical adoption.
Workflow	Workflow integration, interoperability and regulatory requirements	A comprehensive AI ecosystem for POCUS should streamline clinical workflows through seamless automation and integration, improving efficiency while minimizing clinician burden. AI tools should conform to interoperability standards.
Research	Data standardization, integration and regulatory requirements	Standardized AI tools can streamline data curation, improve data quality, enable multicenter collaboration, and accelerate the generation of high-quality evidence for POCUS.
Administration	Generalizability, institutional variability and integration	AI tools can facilitate POCUS program administration by automating quality assurance, compliance monitoring and performance reporting; provided solutions should be adaptable across diverse institutional workflows.

POCUS, point-of-care ultrasound; AI, artificial intelligence.

## 4 Recommendations for the Development Process for POCUS AI Solutions

Adoption of POCUS AI is more likely to be successful if the previously described POCUS AI domains are used to guide strategic development. After choosing a specific domain, we propose that developers follow an 8-step process to maximize the impact of POCUS AI products and improve adoption.

### 4.1 Start with a gap analysis and define outcome objectives

**Figure 1:**
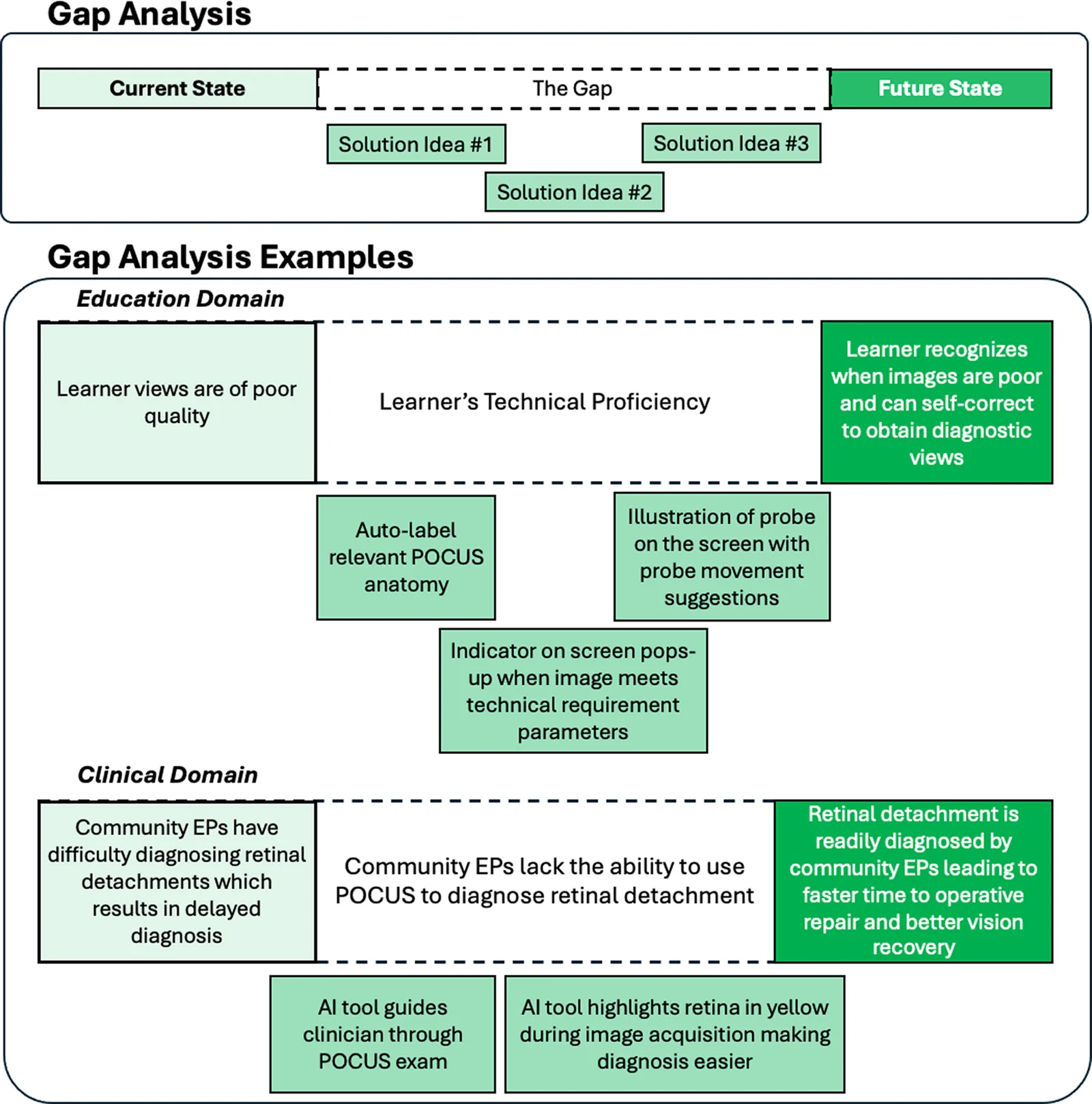
Gap analysis for point-of-care ultrasound developers. EP, emergency physician; POCUS, point-of-care ultrasound; AI, artificial intelligence.

Successful POCUS AI products should begin with an outcomes-based, problem-driven strategy. Developers should collaborate with POCUS leaders to identify relevant and actionable outcome gaps. Clear and specific outcome metrics should be established and agreed upon before the development work begins (Fig 1). Achieving this requires coordinated efforts among clinicians, educators, administrators, and industry to prioritize patient- and system-level outcomes based on the specific development domain.

### 4.2 Consider all POCUS domains and prioritize areas of greatest impact

The POCUS development domains (Table 1) create a holistic approach to understanding POCUS AI use. AI development should focus on domains where the gap between current practice and desired outcomes is greatest. Within clinical care, current evidence suggests that cardiac and lung applications are among the most mature, already demonstrating accurate ejection-fraction estimates and B-line counts. Moving forward, developers should collaborate with the POCUS community to prioritize high-impact, clinically meaningful tools that can transform practice by improving image acquisition, enhancing interpretation, and integrating results into clinical decision-making protocols.

For POCUS AI education, developers should design AI tools that address known training gaps and reduce faculty burden by automating foundational skill training (e.g., interactive guidance of probe position, view recognition, and anatomy identification). This would enable faculty to concentrate on higher-order teaching, such as clinical reasoning and decision-making. Successful AI products should be designed for scalability and accessibility, ensuring training support for clinicians and other health care professionals.

### 4.3 Design for seamless integration into clinical workflows

Developers should ensure that AI tools seamlessly integrate into existing clinical workflows rather than introduce additional steps or administrative burden. High-priority features include automated image labeling, structured reporting, and EHR integration to reduce administrative workload and errors. AI systems should also support quality assurance and billing and reimbursement processes by automatically coding exams and capturing procedural details needed for compliance and financial sustainability. Real-time guidance during image acquisition should be built into workflows to improve efficiency and accelerate clinical decision-making. Developers should emphasize interoperability and standardization so that AI tools function consistently across different machines, clinical environments, operator skill levels, and health information systems. Critically, workflow-focused tools should be evaluated not only on accuracy but also on their ability to reduce time-to-diagnosis, improve efficiency, and support accreditation and quality assurance requirements.

### 4.4 Design data strategy based on target users

Dataset composition must be diverse and aligned with the intended clinical context, including images acquired by both novice and expert sonographers and spanning a wide range of image quality. This allows AI models to recognize common acquisition errors and provide corrective feedback while also learning from high-quality images to optimize performance in expert hands.

The dramatic drop in accuracy when models are applied to new sites underscores the need for multisite data collection, including diverse patient demographics and operator expertise. Transparent documentation of dataset sources and model performance across subgroups will build trust among clinicians and regulators and enable clinicians to judge when and how a model should be applied to specific patient groups and settings.

### 4.5 Develop outputs and interfaces that inspire confidence and adoption

Real-time decision support should be embedded directly into the POCUS interface rather than as a separate application. For acquisition guidance, intuitive visual cues (arrows, color bars) help novices adjust probe position, while automatic quality scoring and “auto-capture” features reduce cognitive load. For interpretation, AI programs should provide an option for explainable outputs (e.g., annotated images showing the region of interest or a plot of ejection-fraction calculation) along with confidence measures. This will enhance adoption of the model. Developers should test interfaces with end users early, iterating based on feedback to ensure that displays improve workflow and do not distract during critical care.

### 4.6 Adopt implementation science and change-management principles

Effective scaling of AI in clinical environments requires structured change management. Initial implementation should begin with pilot programs in select departments, starting with lower-complexity tasks before moving to more complex diagnostic support. Baseline performance metrics should be established and change monitored to ensure improvement in workflow, accuracy, and patient outcomes using established implementation frameworks such as the Consolidated Framework for Implementation Research. A robust education and training program is essential for adoption. Institutions should provide ongoing support and create a culture that views AI as a tool that augments, rather than replaces, clinician judgment. Feedback loops should identify unintended consequences (e.g., over-reliance on AI or alert fatigue) and guide iterative improvement.

### 4.7 Codevelop with stakeholders and integrate AI with automation

End-user-centered development means involving clinicians, educators, researchers, and administrators as indicated throughout the design process. Codevelopment workshops can generate detailed user requirements and identify workflow obstacles. Collaboration between industry, POCUS experts, and health care organizations is essential to ensure that AI algorithms are seamlessly integrated into POCUS hardware and that vendor risk-management standards (security, fairness, and accountability) are met. Standardized data-sharing agreements across institutions can facilitate multicenter datasets and reproducibility. Finally, AI should be combined with simpler automation tools (e.g., rule-based measurement and labeling features) into a single platform. Automation can handle repetitive tasks reliably (e.g., edge detection for chamber measurements), whereas AI provides adaptive interpretation. Integrating both enables a continuum from simple to complex functions, supporting novices while still benefiting experts.

### 4.8 Prepare for regulatory and reimbursement pathways

Developers should engage early with regulators to clarify requirements and ensure patient safety. Regulatory approval of AI algorithms for specific indications (e.g., auto-LVEF and B-line counter) has already been obtained, demonstrating feasibility. For future products, comprehensive validation, documentation of training data, and postmarket surveillance will be required. The newly deployed United States Food and Drug Administration (FDA) program that allows prescriptive algorithm iteration and updating without full 510k resubmission can save developers months to years in updating models but must be worked out with the FDA before receiving clearance. Developers should work with stakeholders to plan for reimbursement mechanisms, as current billing pathways for ultrasound may not recognize AI contributions. Engaging with payers to demonstrate cost-effectiveness and improved outcomes will support long-term adoption. Standardized frameworks should be established to address data privacy, bias mitigation, and liability.

Many POCUS AI applications that are submitted to the FDA for clearance will follow the Software as a Medical Device (SaMD) pathway through the regulatory process. This is a term the FDA defines as "**software intended to be used for one or more medical purposes that perform these purposes without being part of a hardware medical device."** The definition raises an important distinction, that the software is not being submitted as part of a hardware medical device. However, such software is regulated just as any hardware device would be, and thus, falls under 1 of 3 different risk categories, denoted as Class I, II, or III. The majority of POCUS AI applications going through SaMD will fall in the moderate risk category or Class II. Such devices will need to show substantial equivalence with an existing device or predicate for 510(k) clearance. SaMD AI apps that cannot identify a predicate will need to follow the De Novo pathway, which significantly increases time to clearance and overall cost of the process for developers. SaMD software with higher risk, potentially causing harm if it malfunctions, falls under Class III and requires full Premarket Approval under FDA rules. Currently, the FDA is not allowing most POCUS AI applications to make final diagnostic or therapeutic decisions, keeping them out of the Class III category, for now. Validation for 510(k) clearance of POCUS AI will typically require a prospective Pivotal study testing the software use in patients. The rigidity of the testing required varies, based on the AI application itself, and any company progressing to this stage should seek FDA agreement on study design before Pivotal study initiation, typically through a presubmission process. Study sample sizes, number of center locations, and other details have ranged significantly depending on the nature of the AI application itself and the FDA’s view of potential risk of inadequate validation. The agency often requires that the majority of Pivotal study data come from the United States and, in certain instances, insists on geographically distinct study center locations to capture different medical practice locations. Additionally, FDA has specific quality system regulations for any software that handles medical data, such as images, and may interface with medical records. These regulations are well developed and encoded and can be adjusted to keep up with changes in both software development as well as newly recognized potential risks to SaMD products. The emphasis on Quality Management systems and, in recent years, cyber security has meant even small startups have to invest in additional infrastructure and security protocols. Cyber security, especially, has become more complex and highly scrutinized by the agency, leading to proliferation of cyber security companies providing services for SaMD developers, but simultaneously driving up overall development costs.

**Figure 2:**
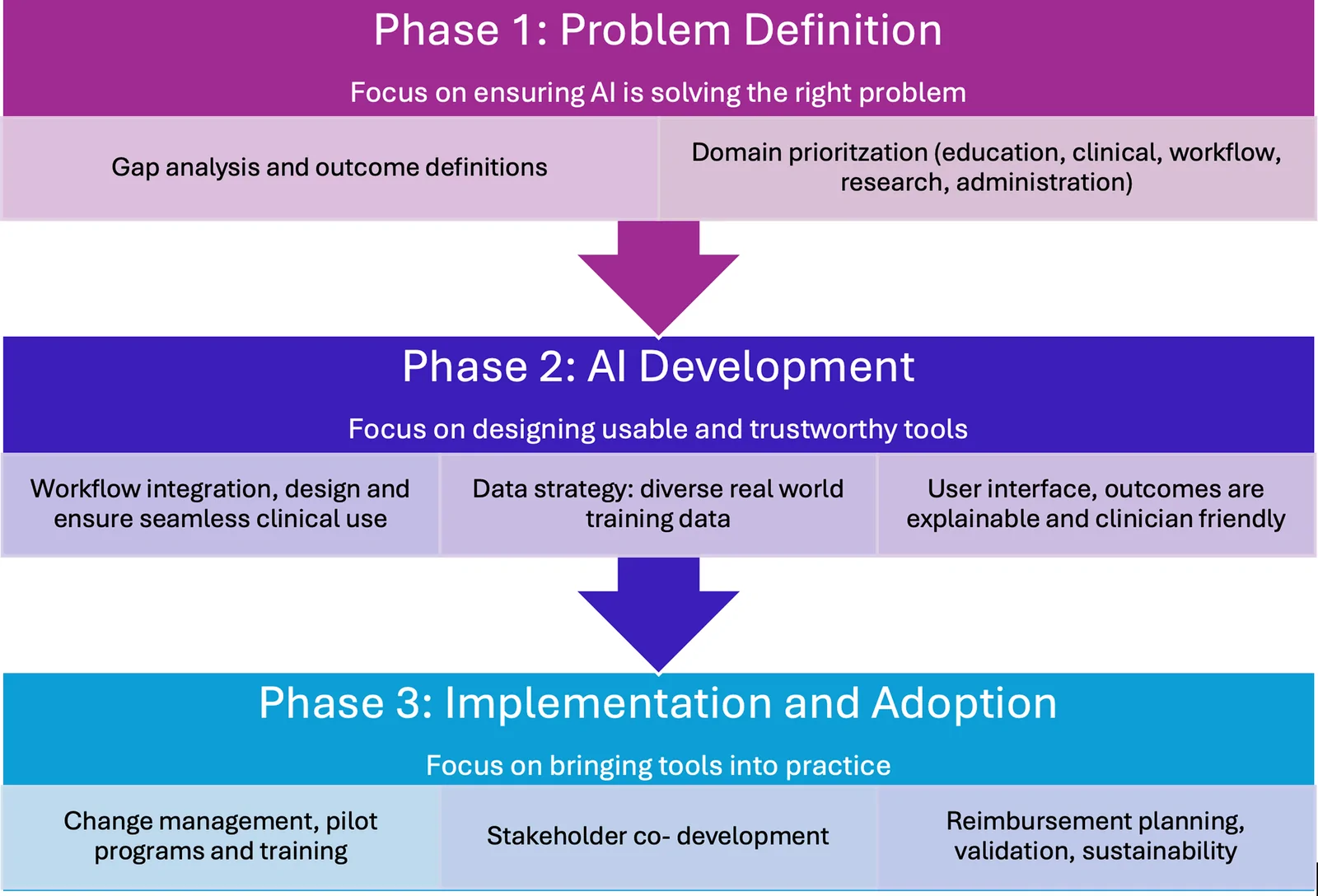
Deliberate approach to the design, development, and implementation of artificial intelligence applications for point-of-care ultrasound.

By following a deliberate process as defined in the steps above, robust POCUS AI products that are trusted and seen as useful solutions are more likely to be adopted within current workflows by intended end users (Fig 2).

## 5 Funding The Development of POCUS AI: Attracting Investors

The development of any POCUS AI product is resource intensive in both time and cost. Although a broad swath of POCUS users may desire POCUS AI tools from a particular domain, it may be unattractive to investors who are needed to pay for the development and/or regulatory costs associated with bringing the product to market. Understanding the funding environment faced by POCUS AI developers is important because investment incentives often shape which technologies are ultimately developed and brought to market. Although clinicians typically prioritize tools that improve patient care, workflow efficiency, or educational outcomes, investors often evaluate opportunities based on scalability, market size, and potential financial return. As a result, the priorities of investors can influence which types of POCUS AI products are pursued and commercialized.

**Figure 3:**
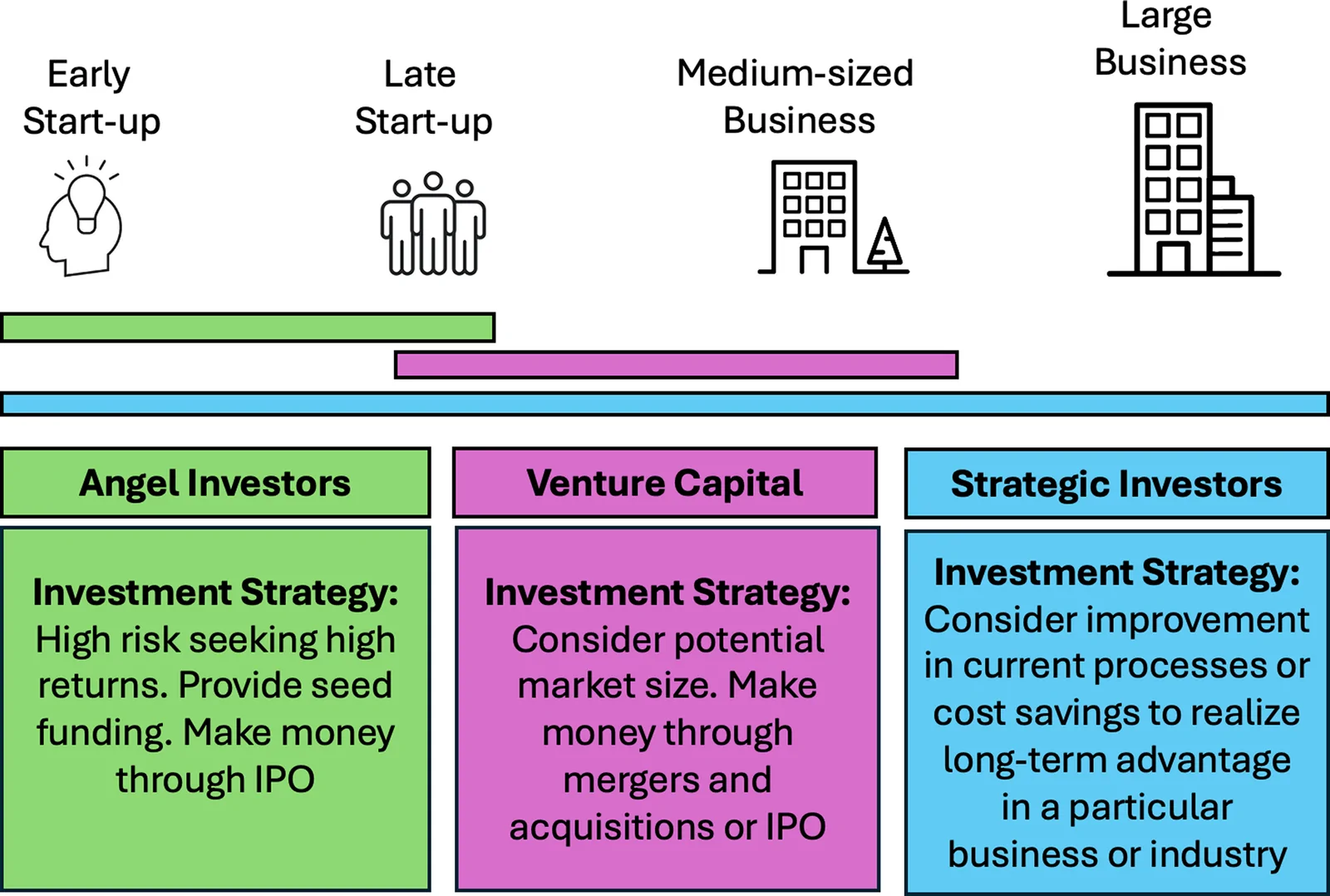
Simplified investor and investment strategy for point-of-care ultrasound artificial intelligence product development. IPO, initial public offering.

Private investment, outside of government and granting agencies, comes in 3 main types of investors: angel, venture capital (VC), and strategic (Fig 3). The angel investor funds early-phase companies to support innovation and may help startups with “seed” rounds or early funding. VC investors generally focus on total market size for the technology to limit their risk and maximize their potential return. Because most of their picks will fail, the few successful ones must generate substantial returns to offset those losses. The lowest risk for investors is a product cleared by the FDA and already selling (an expensive stage to reach), forcing startups to be selective regarding their product and market choice.

Strategic investors are less concerned with revenue generation but tend to focus on the impact a new technology may have on their existing business. These include large hospital systems, which may value technology that delivers increased efficiency and savings. Likewise, giant health care multinational corporations may see a startup’s technology as complementary to their portfolio, understanding that even a small increase in sales of other products can mean large sums of money earned due to acquisition of the new technology.

Although startups frequently focus on clinical impact, investors focus on commercial value and how revenue generation will occur. Patient outcomes and cost savings are important to investors, but demonstrating this is challenging and adds additional expense, leaving niche investors and strategic investors as the only likely investor candidates. As a result, investment in POCUS AI may remain a challenge until a clear track record of revenue generation, exit pattern, and profit potential is established. For developers and users alike, it is crucial to be selective in choosing POCUS AI solutions that will have both significant impact and commercialization value.

## 6 Developer Guardrails

Developer guardrails for AI programs in POCUS are essential to protect patient safety and prevent use beyond a tool’s validated scope. It is also mandatory for AI developers to comply with existing government rules and regulations in the geographical locations where their models are intended to be deployed.

The scope of the AI model should be clearly defined. Training datasets should represent diverse patient populations and include images of varying quality. Datasets should be free of demographic imbalances that could inadvertently contribute to health care disparities.^35^ Training datasets should also be fully compliant with Health Insurance Portability and Accountability Act standards.

Because AI in ultrasound has the potential to directly influence clinical decision-making, it must be developed with transparency.^36^ Without compromising proprietary information or patient privacy, developers should release the demographic characteristics of patients used for training of their AI models. They should clearly articulate both the reasoning behind model outputs and the degree of certainty associated with their conclusions. In addition, vendors should clearly describe how the model was developed and validated, so clinicians can make informed decisions about when and how to apply it in their specific clinical settings. Whenever possible, limitations of AI models should be clearly stated to minimize risks of generating inaccurate or misleading results.

Following implementation, AI models should undergo ongoing performance audits as part of a structured quality assurance process. A clear mechanism for users to report errors must be established, with the development team remaining actively engaged in refining and improving the system over time.^37^ Institutions should also establish local governance structures to oversee AI deployment and ensure that algorithms continue to perform reliably within the local clinical environment. This includes monitoring performance on local datasets and defining responsibility for postdeployment oversight.

Emerging transparency frameworks, such as Coalition for Health AI’s “AI nutrition labels,” model cards, and related reporting standards, have been proposed to address safety concerns by providing structured documentation of model development, including training data characteristics, performance across populations, known limitations, intended clinical use, and requirements for human oversight. Such approaches may improve transparency and help support safer and more informed clinical adoption of AI systems.^38^^,^^39^

## 7 Conclusion

Despite the growing availability of POCUS AI, adoption remains limited due to misalignment with clinical needs, data limitations, workflow barriers, and insufficient outcome evidence. By framing AI development within clearly defined POCUS AI domains, prioritizing outcome-driven use cases, and engaging stakeholders across clinical, educational, administrative, research, and industry settings, developers can create AI tools that are more relevant, trustworthy, and scalable. Aligning clinical impact with commercial viability and regulatory pathways will sustain innovation. POCUS AI should be viewed as an augmentative technology that, when responsibly developed and deployed, can improve diagnostic accuracy, efficiency, education, and patient-centered care.

## Funding and Support

By *JACEP Open* policy, all authors are required to disclose any and all commercial, financial, and other relationships in any way related to the subject of this article as per ICMJE conflict of interest guidelines (see www.icmje.org). The authors have stated that no such relationships exist. The authors have stated that no such relationships exist.

## Conflict of Interest

Dr Ferre reports a relationship with Butterfly Network, Inc, FujiFilm Sonosite, Inc, and Philips Healthcare: consulting or advisory. Dr Liu reports a relationship with GE Healthcare Technologies, Inc and Butterfly Network, Inc.: consulting or advisory. Dr Blaivis reports a relationship with ThinkSono that includes: consulting or advisory. Dr Bolescu reports receiving research funding Philips Healthcare and Rivanna Medical through Biomedical Advanced Research and Development Authority (BARDA)-funded research agreements, and research funding from GE Healthcare. Dr Bolescu also reports a relationship with GE Healthcare under a BARDA-funded award that includes: consulting or advisory. Dr Adhikari reports a relationship with GE Healthcare, EchoNous, ThinkSono, and Autonomus Medical Technologies Inc: consulting or advisory. All other authors have affirmed they have no conflicts of interest to declare.
